# Emerging roles of the gut microbiota in cancer immunotherapy

**DOI:** 10.3389/fimmu.2023.1139821

**Published:** 2023-02-22

**Authors:** Zhuangzhuang Shi, Hongwen Li, Wenting Song, Zhiyuan Zhou, Zhaoming Li, Mingzhi Zhang

**Affiliations:** ^1^Department of Oncology, The First Affiliated Hospital of Zhengzhou University, Zhengzhou, China; ^2^Lymphoma Diagnosis and Treatment Centre of Henan Province, Zhengzhou, China; ^3^Academy of Medical Sciences of Zhengzhou University, Zhengzhou, Henan, China; ^4^State Key Laboratory of Esophageal Cancer Prevention and Treatment and Henan Key Laboratory for Esophageal Cancer Research, The First Affiliated Hospital of Zhengzhou University, Zhengzhou, Henan, China

**Keywords:** Gut microbiota, immunotherapy, immune checkpoint blockade, allogeneic hematopoietic stem cell transplantation, chimeric antigen receptor T cell therapy

## Abstract

Gut microbiota represents a hidden treasure vault encompassing trillions of microorganisms that inhabit the intestinal epithelial barrier of the host. In the past decade, numerous *in-vitro*, animal and clinical studies have revealed the profound roles of gut microbiota in maintaining the homeostasis of various physiological functions, especially immune modulation, and remarkable differences in the configuration of microbial communities between cancers and healthy individuals. In addition, although considerable efforts have been devoted to cancer treatments, there remain many patients succumb to their disease with the incremental cancer burden worldwide. Nevertheless, compared with the stability of human genome, the plasticity of gut microbiota renders it a promising opportunity for individualized treatment. Meanwhile, burgeoning findings indicate that gut microbiota is involved in close interactions with the outcomes of diverse cancer immunotherapy protocols, including immune checkpoint blockade therapy, allogeneic hematopoietic stem cell transplantation, and chimeric antigen receptor T cell therapy. Here, we reviewed the evidence for the capacity of gut microflora to modulate cancer immunotherapies, and highlighted the opportunities of microbiota-based prognostic prediction, as well as microbiotherapy by targeting the microflora to potentiate anticancer efficacy while attenuating toxicity, which will be pivotal to the development of personalized cancer treatment strategies.

## Introduction

The human gut microbiota refers to the vast collection of various microbes living on the epithelial barrier surfaces of the gastrointestinal tract, including bacteria, fungi, viruses, archaea, and protozoa (1). With the advances of molecular tools and technologies such as 16S ribosomal RNA sequencing, metagenomic, metabolomic, and metatranscriptomic, as well as the use of gnotobiotic animal models, the intricate host-microbiota interactions are progressively being deciphered (2). For one thing, substantial researches have featured the key roles of gut microbiota in human pathophysiological processes (3–5), including immunity, metabolism, and inflammatory response. For another, albeit multiple factors are proposed to propel cancer progression, the deviation of gut microbiota, known as dysbiosis, is entertained as a harbinger, promoter or even cause of a variety of malignant conditions (6). Thereinto, a panel of potential pro-tumorigenesis or anti-tumorigenesis microbial species have been identified too (7), which lays the groundwork for the regulation of gut microbiota in cancer therapy.

Meanwhile, with the incremental cancer burden worldwide, it places greater demands on personalized cancer treatments with powerful efficacy (8), although substantial advancements have been made, especially cancer immunotherapy. Most notably, the limited efficacy and undesired toxicities still remain the major hurdles of current cancer therapies, which has been found to be heavily influenced by distinct gut microflora patterns (9). Of them, immunotherapy has been considered as a major revolution, which provides exciting hopes for patients in the fight against cancer, and the effects of certain gut species on immunotherapy have now become a topic of great scientificity (10, 11). In the light of these findings, there is emerging interest in microbiotherapy by the modulation of intestinal flora as one of the antitumor strategies in recent years.

**Figure 1:**
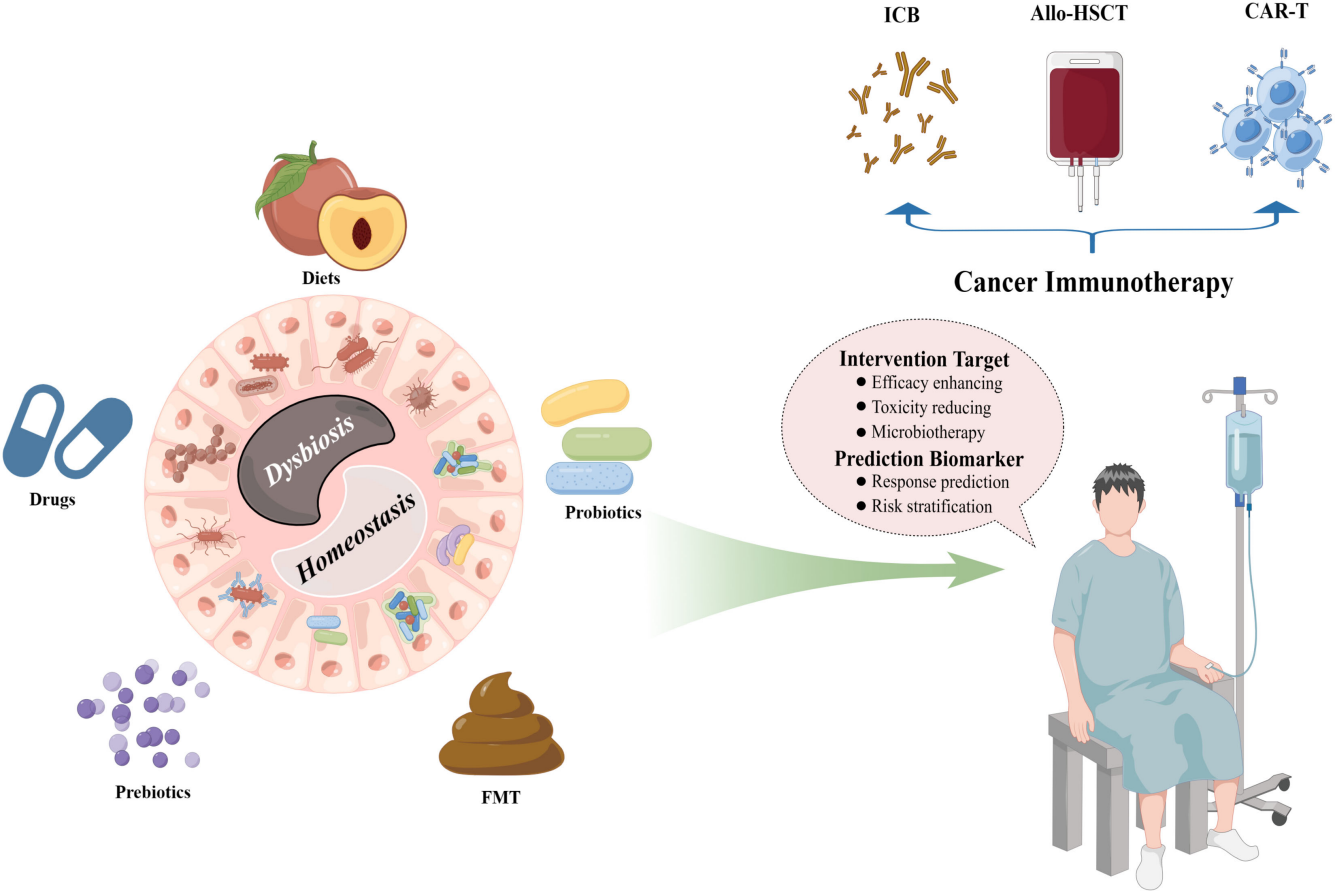
Interactions between the gut microbiota and cancer immunotherapy. The intestinal microbial ecosystem can be well modified by multiple patterns, including diets, drugs, prebiotics, probiotics, and FMT, which provide fascinating opportunities for the clinical managements of diverse cancer immunotherapy protocols such as ICB therapy, allo-HSCT, and CAR-T cell therapy. FMT, fecal microbiota transplantation; ICB, immune checkpoint blockade; allo-HSCT, allogeneic hematopoietic stem cell transplantation; CAR-T, chimeric antigen receptor T. (By Figdraw).

In this review, we mainly discussed the interactions between gut microbiota and cancer immunotherapies, including immune checkpoint blockade (ICB) therapy, allogeneic hematopoietic stem cell transplantation (allo-HSCT), and chimeric antigen receptor T (CAR-T) cell therapy, and the opportunities of microbiota-based patient stratification strategies such as the prediction of response and the early recognition of toxic events, as well as the evidence for the ability of microbiotherapy in the management of cancer immunotherapy, including enhancing anticancer efficacy and alleviating toxicity, thus, to decipher the roadmap of gut microbiota in the exploitation of custom-fit therapeutic strategies for cancer care. A diagrammatic representation of the interactions between gut microbiota and cancer immunotherapy is described in **Figure 1**.

## Immune checkpoint blockade therapy

Currently, one hotspot of cancer immunotherapy is the ICB therapy that inhibits programmed cell death protein 1/programmed cell death ligand 1 (PD-1/PD-L1) and cytotoxic T lymphocyte-associated antigen-4 (CTLA-4) signaling to reinvigorate CD8+ T cells in the tumor microenvironment (TME) to potentiate killing of tumor cells (12). Despite the remarkable effectiveness of ICB therapy in a subset of patients of several cancer types, including metastatic melanoma (13), classical Hodgkin lymphoma (14), non-small cell lung cancer (NSCLC) (15), and colon cancers (16), most patients were observed with primary or acquired resistance. Furthermore, a number of challenges such as the immune-related adverse events (irAEs) and biomarkers to predict response remain to be determined (17). However, accumulating data have pinpointed the indispensable roles of intestinal microbiota in ICB therapy.

### The effects of antibiotics on the response of ICB therapy

Antibiotics-associated gut dysbiosis frequently confers deleterious effects on cancer patients treated with ICB (18). Derosa and colleagues (19) reported that antibiotics administration within 30 days of beginning ICB therapy was closely related to the inferior prognosis, including shorter progression free survival and overall survival (OS), in both advanced renal cell carcinoma and NSCLC patients. Similarly, the negative influences of antibiotics on the clinical outcomes of ICB have also be indicated in patients with melanoma (20), urothelial carcinoma (21), and bladder cancer (22). Nevertheless, Cheung et al. (23) and Fessas et al. (24) inversely revealed the detrimental and protective effects of antibiotics use on the survival of ICB treated hepatocellular carcinoma patients, respectively, which might be attributable to the difference of the antibiotic types, therapeutic regimens, baseline clinical characteristics and gut microbial features of patients. In addition, one caveat here is the antibiotics application might simply constitute a surrogate indicator of unsuited or immunodeficient cancer patients who were non-responsive for ICB therapy, which deserves further evaluation.

### Influence of gut microbiota on the effectiveness of ICB therapy

The significance of commensal intestinal bacteria on the efficacy of ICB therapy has been well established in both pre-clinical models and patients. A plethora of microbial taxa, including *Akkermansia muciniphila*, *Faecalibacterium* spp., *Bifidobacterium* spp., and *Bacteroides fragilis* (25), have been reported to potentiate the antitumor efficacy of ICB therapy in both animal models and cancer patients. Of specific note, it has been well demonstrated that gut commensal bacteria such as *Bifidobacterium* and *Bacteroidales* could significantly improve tumor control of melanoma treated by anti-PD-L1 or anti-CTLA-4 *via* enhancing antitumor immunity response in mice models (26, 27). Further, this favorable role of commensal microbiome in ICB therapy was elucidated in melanoma patients (28, 29). Additionally, a higher diversity of gut microbiota at the starting point exhibited intimate relationships with the favorable responses to anti-PD-1 immunotherapy in patients with hepatocellular carcinoma and advanced NSCLC (30, 31), which might be involved in the enhanced memory T cell and natural killer cell signatures in the periphery in response to anti-PD-1 therapy. Interestingly, *Helicobacter pylori* seropositivity has been reported to be linked with an inferior NSCLC patient survival on anti-PD-1 therapy (32), and further be confirmed in *in vitro* co-culture assay and in *H. pylori*-infected mice with reduced number and activation status of tumor-specific CD8+ T cells in the tumors. Strikingly, apart from the linkages between individual bacterial taxa and ICB therapy outcome, the association of enteric microbiotypes (including diverse discrete ecologically balanced communities) with the response to melanoma patients treated by anti-PD-1 has also been proposed in a recent combination analysis (33). That is, four superclusters of a panel of microbial species, including two enriched in favorable taxa (Favorable 1: *Bifidobacteriaceae*, *Eggerthelacea*, *Coriobacteriales*, *Akkermansia muciniphila*, *Fusobacteriaceae*, *Erysipelotrichaceae*, *Lachnospiraceae*, *Streptococcaceae*, *Lactobacillaceae*, and *Porphyromonadaceae*; Favorable 2: *Oscillospiraceae*; by linear discriminant analyses) and two enriched in unfavorable taxa (Unfavorable 1: *Prevotellaceae* and *Bacteroidales*; Unfavorable 2: *Rikenellaceae*; by linear discriminant analyses), were defined, which comprised distinct microbiotypes with similar relationship between microbial composition and clinical outcome.

Various publications have now demonstrated a role for gut microbes in regulating responses to ICB therapy across several cancer types (**Table 1**). In a phase I clinical trial including ten patients with anti-PD-1-refractory metastatic melanoma (52), the researchers found that re-induction of anti-PD-1 combination with fecal microbiota transplantation (FMT) from complete response donors exhibited inspiring outcomes with clinical remission in three patients. Of them, FMT could remarkably increase the intra-tumoral immune activity, which supports the concept of overcoming resistance to immunotherapy through manipulating the intestinal microflora. Another multicenter retrospective study from Japan (53) revealed that probiotics administration was relevant to the survival and disease control in advanced or recurrent NSCLC patients that undergone anti-PD-1 monotherapy. Despite this, more thoughtful evaluations of the effects of current commercially available probiotic formulations on anticancer immunotherapy should be made, as they might be harmful in the setting of ICB therapy by impairing intra-tumoral IFN-γ T cell responses (40).

**Table 1:** Representative researches on the interactions between gut microbiota and the outcomes of ICB therapy across cancers in recent three years.

Patients	Studies	ICB agents	Main findings
**NSCLC**	Grenda et al. (34)	Pembrolizumab, n = 12, 25%; nivolumab or atezolizumab, n = 35, 75%	Favorable survival: a high abundance of *Bacteroidaaceae*, *Barnesiellaceae*, and *Tannerellaceae*; Inferior survival: a high content of *Ruminococcaceae* family while a low abundance of *Clostridia UCG-014*.
	Shoji et al. (35)	Nivolumab, pembrolizumab, atezolizumab, or durvalumab, n = 24, 85.7%; pembrolizumab combined with platinum-doublet chemotherapy, n = 4, 14.3%	Responders: higher gut alpha diversity; enrichment of *g_Blautia*; Non-responders: enrichment of *o_RF32* order.
	Newsome et al. (36)	Anti-PD-1, n = 44, 67.7%; anti-PD-L1, n = 19, 29.2%; combination of anti-PD-L1/CTLA-4, n = 2, 3.1%	Responders: enrichment of the genera *Ruminococcus*, *Akkermansia*, and *Faecalibacterium*.
	Zhang et al. (37)	A total of 69 patients receiving ICB monotherapy, including nivolumab, pembrolizumab, or atezolizumab	Prolonged survival: enrichment of *Phascolarctobacterium*; Reduced survival: overrepresentation of *Dialister*.
	Zhang et al. (38)	Nivolumab, n = 36, 48.0%; pembrolumab, n = 39, 52.0%	Responders: higher gut microbiota alpha diversity; enrichment of *Desulfovibrio*, *Actinomycetales*, *Bifidobacterium*, *Odoribacteraceae*, *Anaerostipes*, *Rikenellaceae*, *Faecalibacterium*, and *Alistipes*; Non-responders: overrepresentation of *Fusobacterales*, *Fusobacteriia*, *Fusobacterium*, *Fusobacteria*, and *Fusobacteriaceae*.
	Botticelli et al. (39)	Nivolumab, n =12, 100%	Clinical benefits: short chain fatty acids (i.e., propionate, butyrate), lysine and nicotinic acid were significantly associated with long-term beneficial effects; Disease progression: 2-Pentanone (ketone) and tridecane (alkane) were significantly associated with early progression.
**Melanoma**	McCulloch et al. (33)	A total of 94 patients receiving single-agent anti-PD-1 immunotherapy, including nivolumab, pembrolizumab or investigational anti-PD-1, or pembrolizumab in combination with pegylated interferon	Non-progressors: enrichment of *Ruminococcus* (*Mediterraneibacter*) *torques*, *Blautia producta*, *Blautia wexlerae*, *Blautia hansenii*, *Eubacterium rectale*, *Ruminococcus* (*Mediterraneibacter*) *gnavus* and *Anaerostipes hadrus*; Progressors: increased abundance of *Prevotella* spp., *Oscillibacter* spp., *Alistipes* spp. and *Sutterellaceae* spp.
	Spencer et al. (40)	Anti-PD-1, n = 132, 100%	Responders: higher abundance of *Ruminococcaceae* family and *Faecalibacterium* genus than non-responders.
	Andrews et al. (41)	A total of 77 patients receiving ipilimumab (anti-CTLA-4) in combination with PD-1 checkpoint blockade agent (either nivolumab or pembrolizumab)	Responders: enrichment of *Bacteroides stercoris*, *Parabacteroides distasonis* and *Fournierella massiliensis*; Non-responders: overrepresentation of *Klebsiella aerogenes* and *Lactobacillus rogosae*.
**HCC**	Lee et al. (42)	Nivolumab, n = 24, 58.5%; pembrolizumab, n = 17, 41.5%	Responders: enrichment of *Lachnoclostridium*, *Lachnospiraceae*, and *Veillonella*; Disease progression: overrepresentation of *Prevotella 9*.
	Wu et al. (43)	A total of 61 patients receiving intravenously anti-PD-1 based systemic therapy	Responders: enrichment of *Faecalibacterium*, *Blautia*, *Lachnospiracea incertae* Sedis, *Megamonas*, *Ruminococcus*, *Coprococcus*, *Dorea* and *Haemophilus*; Non-responders: overrepresentation of *Atopobium*, *Leptotrichia*, *Campylobacter*, *Allisonella*, *Methanobrevibacter*, *Parabacteroides*, *Bifidobacterium* and *Lactobacillus*.
	Ponziani et al. (44)	A total of 11 patients received tremelimumab, an anti-CTLA-4 monoclonal antibody, and/or Durvalumab, an anti-PD-L1 monoclonal antibody	Responders: enrichment of *Akkermansia* whereas depletion of *Enterobacteriaceae* in disease control group versus non-responders.
**HBC**	Mao et al. (45)	Thirty patients with HCC and 35 patients with biliary tract cancer who were treated with anti-PD-1 based systemic therapy	Favorable survival: enrichment of *Lachnospiraceae bacterium-GAM79*, *Alistipes sp Marseille-P5997*, *Ruminococcus calidus*, and *Erysipelotichaceae bacterium-GAM147*; Worse survival: higher abundance of *Veillonellaceae*.
**CRC**	Wang et al. (46)	Phase Ib/II study of regorafenib plus toripalimab enrolled forty-two subjects	Non-responders: increased relative abundance and positive detection rate of *Fusobacterium* than responders.
**GC**	Che et al. (47)	Nivolumab, n = 43, 55.8%; pembrolizumab, n = 29, 37.7%; camrelizumab/toripalimab/tislelizumab, n = 5, 6.5%	*Helicobacter pylori*-negative group: a longer overall survival (OS) and progression-free survival (PFS) than those in the positive group, with an estimated median OS of 17.5 months vs. 6.2 months (HR = 2.85, 95% CI: 1.70-4.78; *P* = 0.021) and a median PFS of 8.4 months vs. 2.7 months (HR = 3.11, 95% CI: 1.96-5.07, *P* = 0.008); *H. pylori*-positive group: a higher risk of nonclinical response to anti-PD-1 antibody, with an OR of 2.91 (95% CI: 1.13-7.50).
**GI cancer**	Peng et al. (48)	Anti-PD-1, n = 48, 64.9%; anti-PD-L1, n = 12, 16.2%; anti-PD-1 + anti-CTLA-4, n = 14, 18.9%	Responders: an elevation of the *Prevotella*/*Bacteroides* ratio; moreover, gut bacteria with the ability of SCFA production, including *Eubacterium*, *Lactobacillus*, and *Streptococcus*, were positively associated with anti-PD-1/PD-L1 response across different GI cancer types (colorectal cancer, n = 19; esophageal cancer, n = 14; gastric cancer, n = 23; Others, n = 18).
**RCC**	Salgia et al. (49)	Nivolumab, n = 24, 77.4%; nivolumab plus ipilimumab, n = 7, 22.6%	Clinical benefits: a higher gut microbial alpha diversity according to the Shannon index; enrichment of *Bifidobacterium adolescentis*, *Barnesiella intestinihominis*, *Odoribacter splanchnicus*, and *Bacteroides eggerthii*.
**Thoracic carcinoma^※^**	Yin et al. (50)	A total of 42 patients receiving nivolumab or other anti-PD-1 inhibitors	Responders: enrichment of the *Akkermansiaceae*, *Enterococcaceae*, *Enterobacteriaceae*, *Carnobacteriaceae* and *Clostridiales Family XI* bacterial families.
**Solid cancer tumors^#^**	Cheng et al. (51)	A total of 72 patients receiving nivolumab, pembrolizumab, sintilimab, camrelizumab, and toripalimab	Responders: enrichment of *Archaea*, *Lentisphaerae*, *Victivallaceae*, *Victivallales*, *Lentisphaeria*, *Methanobacteriaceae*, *Methanobacteria*, *Euryarchaeota*, *Methanobrevibacter*, and *Methanobacteriales* before immunotherapy; Non-responders: increased in the abundance of *Clostridiaceae* before immunotherapy.

ICB, immune checkpoint blockade; NSCLC, non-small cell lung cancer; HCC, hepatocellular carcinoma; HBC, hepatobiliary cancer; CRC, colorectal cancer; GC, gastric cancer; GI, gastrointestinal; RCC, renal cell carcinoma; PD-1, programmed cell death protein 1; PD-L1, programmed cell death ligand 1; CTLA-4, cytotoxic T lymphocyte-associated antigen-4; OS, overall survival; PFS, progression-free survival. ※: included 23 lung squamous carcinomas, 15 lung adenocarcinomas, 1 SCLC, 1 NSCLC, 1 thymic squamous carcinoma, and 1 large cell neuroendocrine carcinoma; #: included 18 non-squamous NSCLC, 14 lung squamous cell carcinoma, 7 HCC, 5 GC, 5 CRC, 5 melanoma, 4 nasopharyngeal carcinoma, 3 cervical cancer, 2 small-cell lung cancer, and other cancers (1 case of laryngeal cancer, 1 case of osteosarcoma, 1 case of renal pelvic carcinoma, 1 case of bladder cancer, 1 case of pancreatic cancer, 1 case of esophageal cancer, 1 case of ureteral cancer, 1 case of mediastinal carcinoma, and 1 case of cholangiocarcinoma).

### Interactions of gut microbiota with the toxicities related to ICB therapy

Evidence is accumulating that certain fecal microbiota composition is related to the development of several toxicities following ICB therapy such as irAEs, which result from off-tumor immune activation. McCulloch et al. (33) indicated that two microbial signatures, enriched for *Streptococcaceae* spp. and *Lachnospiraceae* spp., were involved in distinct irAEs, and melanoma patients with high *Streptococcus* spp. abundance in pretreatment microbiome samples tended to develop irAEs. Although higher rates of irAEs than anti-PD-1 or anti-CTLA-4 monotherapy, responders to combined ICB therapy targeting both CTLA-4 and PD-1 and responders to monotherapy exhibited similar compositional characteristics of gut microbiota with an enrichment of *Ruminococcus*/*Ruminococcaceae* consistently observed across diverse melanoma cohorts (41). Moreover, the researchers found a significantly higher abundance of *Bacteroides intestinalis* in patients developed ≥grade 3 irAEs versus those who did not, with upregulation of mucosal IL-1β in patient samples of colitis and in pre-clinical models.

Disturbances of intestinal homeostasis play a key role in driving ICB-associated toxicity. Stat3^+/+^ melanoma-bearing mice with acquired gastrointestinal impairment by *Citrobacter rodentium* infection and dextran sodium sulfate treatment displayed a predilection for anti-CTLA-4-mediated irAEs, with accumulation of neutrophils, cytotoxic and IFN-γ+ CD8+ and CD4+ T cells, and inflammatory cytokines such as IFN-γ and IL-6 in the colon (54). Furthermore, the pre-inflammation fecal microbiota of melanoma patients that presented a paucity of genetic pathways related to polyamine transport and B vitamin biosynthesis was linked with an increased risk of colitis (55). Remarkably, modulation of the gut microbiota can mitigate irAEs in cancers (56). Of them, ICB-related colitis could be successfully treated by FMT, with reconstitution of the intestinal microflora and increase in the proportion of regulatory T cells within the colonic mucosa (57). Additionally, microbial metabolites working at the interface between microorganisms and host immune system might abrogate ICB-induced colitis too. Renga et al. demonstrated that indole-3-carboxaldehyde, a microbial tryptophan catabolite, protected ICB-induced colitis mice from intestinal injury through a dual action on both the host and the microbes (58), which provides a new avenue in optimizing ICB therapy based on bacterial metabolome.

### Prognostic utility of the gut microbiota-derived models for the outcome of ICB therapy

Gut microflora has emerged as a tumor-extrinsic predictive biomarker to the response of ICB therapy, and the machine learning models trained by microbial features provide a hopeful opportunity for outcome prediction. Recently, despite the heterogeneity across five melanoma cohorts, three modified leave-one-out cross-validation methods, including generalized linear model, random forest, and polynomial support-vector machine, based on batch-corrected intestinal microbiome data consistently predicted the outcomes to anti-PD-1 therapy in all cohorts (33). Of them, the *Clostridium* phylum was identified as a predictor of favorable outcome for a subset of cohorts, while the *Bacteroidetes* phylum was entertained as an unfavorable predictive indicator for the response of most melanoma cohorts. In addition, based on the bacterial signatures of five cancer cohorts, including melanoma, NSCLC, and renal cell carcinoma, treated with ICB, Shaikh et al. (59) constructed a non-responder “Integrated Microbiome Prediction Index” (calculated by assigning a weighted coefficient for the microbial species enriched in non-responders, including *Bacteroides coprocola*, *Bacteroides fragilis*, *Bacteroides thetaiotaomicron*, *Bacteroides uniformis*, *Clostridium hathewayi*, *Clostridium hylemonae*, *Clostridium methylpentosum*, *Megasphaera micronuciformis*, *Oribacterium sinus*, *Parasutterella excrementihominis*, *Scardovia wiggsiae*, and *Veillonella parvula*), rather than responder, that displayed the strongest and most consistent signal using a random effects model, which highlighted a novel avenue to recognize specific patients that probably benefit from microbiota-derived interventions to improve the outcomes of ICB therapy.

### Gut microbiota-derived metabolites mediate the responses of ICB therapy

**Figure 2:**
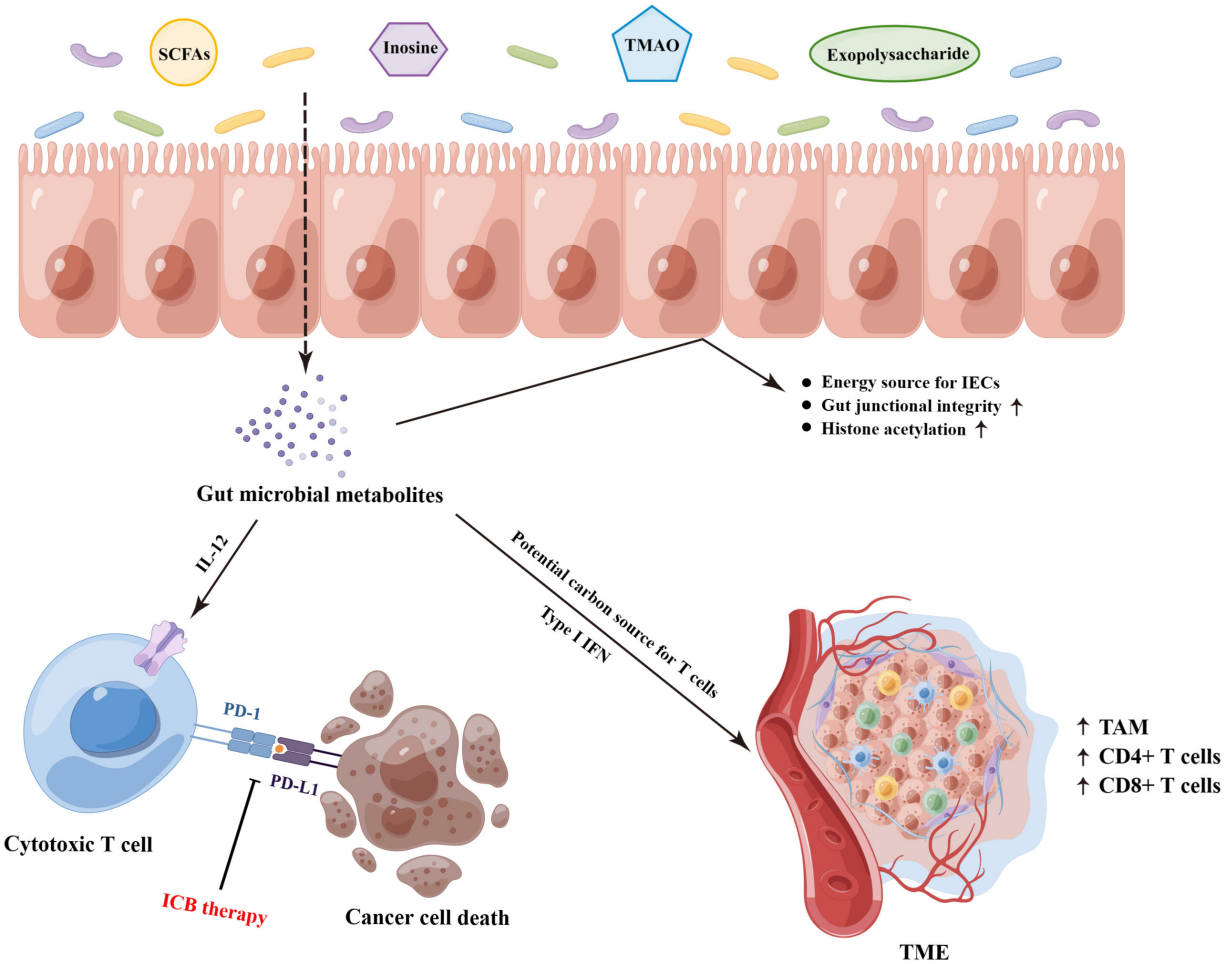
The underlying molecular mechanisms on the gut microbiota-derived metabolites that mediated the responses of ICB therapy. SCFAs, short−chain fatty acids; TMAO, trimethylamine N-oxide; IECs, intestinal epithelial cells; ICB, immune checkpoint blockade; TAM, tumor-associated macrophage; TME, tumor microenvironment. (By Figdraw).

The gut microbial metabolites, a vast array of small molecules produced or transformed by intestinal microorganisms, represent one of the primary patterns by which the gut microbiota regulate antitumor immunity response, which are capable of conferring both local and systemic effects by spreading from their original location in the gastrointestinal tract to circulatory system (10, 60). Accordingly, it is necessary to dissect the underlying mechanistic pathways through which the specific bacterial metabolites impact on antitumor immunity and immunotherapeutic responses (**Figure 2**). Most notably, short−chain fatty acids (SCFAs, mainly including acetate, propionate, and butyrate), synthetized by the bacterial fermentation of dietary fiber, play a central role in the complicated gut microbial immune and metabolic networks (61). Of them, the gut microbial metabolite butyrate has been reported to engage in the enhanced anti-PD-1 therapeutic efficacy through increasing the CD4+ and CD8+ T cell infiltration in the TME in the tumor-bearing mice humanized with the intestinal microbes from colorectal cancer (CRC) patients (62). Moreover, replenishing butyrate prior to anti-PD-1 treatment was sufficient to recover the therapeutic efficacy in the non-responders. Similarly, He and colleagues indicated that the SCFAs butyrate could directly potentiate the antitumor CD8+ T cell response *via* ID2-dependent IL-12 signaling (63), suggesting the potential beneficial roles of butyrate supplementation in anticancer immunity therapy. However, the roles of SCFAs in ICB therapy might be ambiguous. In a study conducted by Coutzac and colleagues in both mice and melanoma patients treated with anti-CTLA-4 monoclonal antibody, the authors found that SCFAs limited the efficacy of anti-CTLA-4 treatment. Namely, high levels of blood butyrate and propionate were involved in the resistance to CTLA-4 blockade and higher frequency of Treg cells, and butyrate could impede the accumulation of tumor-specific T cells and memory T cells (64). Therefore, further evaluations are warranted to reveal a more nuanced illustration for the effects of SCFAs on antitumor immune and the responses to ICB therapy.

In addition to SCFAs, other gut microbial metabolites also exhibit profound effects on the treatment of ICB. Strikingly, the bacterial metabolite inosine has been demonstrated to modulate enhanced ICB therapy response in mouse models of intestinal and epithelial tumors, which was dependent on T cell expression of the adenosine A_2A_ receptor to promote Th1 cell activation (65). Furthermore, as a substitute carbon source for the metabolism of T lymphocyte in glucose-restricted environments such as TME, inosine can assist T cell proliferation and differentiation while fueling sensitivity to ICB therapy (66). Another gut microbiota-derived metabolite trimethylamine N-oxide, identified as a driver of antitumor immunity, exhibited the ability to boost the response to ICB therapy in pancreatic cancer-bearing mouse model (67). Of them, the administration of trimethylamine N-oxide was related to an immunostimulatory tumor-associated macrophage phenotype, and activated effector T cell response in the TME in a type I IFN-dependent manner. Interestingly, Kawanabe-Matsuda et al. (68) illustrated that oral consumption of *Lactobacillus*-derived exopolysaccharide could bolster the efficacy of ICB therapy against CCL20-expressing tumors *via* inducing CCR6+ CD8+ T cells in Peyer’s patches and improving the TME in experimental mouse tumor models, which provided compelling evidence on the dietary ingestion of exopolysaccharide for further clinical trials. Altogether, these studies lay the groundwork for the potential cancer immunotherapeutic strategies by targeting gut microbiota-derived metabolites.

## Allogeneic hematopoietic stem cell transplantation

Allo-HSCT remains a curative approach for a range of hematological malignancies and might be recognized as one of the earliest effective modalities for cancer immunotherapy, but it is still hindered by high mortality rates, mainly because of graft-versus-host disease (GVHD) (69). Notably, at present the interactions between the intestinal microbiota and patient outcome after allo-HSCT have been well established (70). Particularly, ample evidence indicates an effect of gut microbial dysbiosis on GVHD. Furthermore, gut microbiota modulation through FMT also exhibits a promising revolution in the managements of allo-HSCT recipients, including ameliorating treatment-associated complications and improving patient outcomes.

### Relationships between gut microbiota and allo-HSCT

Holler et al. (71) conducted a prospective research to collect stool specimens from 31 patients receiving allo-HSCT, and revealed that the loss of bacterial diversity and predominance of *enterococci* induced by systemic antibiotics might involve in the pronounced gastrointestinal GVHD for the first time. The patterns of microbial dysregulation during allo-HSCT were similar across diverse transplantation centers and geographic locations, and the depletion of gut diversity during allo-HSCT (accompanied by the domination of single taxa such as the genera *enterococcus* and *streptococcus*) has been observed to be linked with higher risks of transplantation-associated death in a large multi-center study (72). Recently, Andrlová and colleagues (73) indicated that a diverse gut microbiota early after allo-HSCT could produce more activating ligands for innate-like mucosal-associated invariant T cells and Vδ2 cells to maintain the immunological link between these populations, which contributed to improved OS and less acute GVHD. Furthermore, *Enterococcal* expansion after allo-HSCT as a remarkable risk factor for the occurrence of acute GVHD and reduced OS has been observed again in another multi-center study including 1325 recipients, and further be demonstrated in mouse models (74). Moreover, the researchers also found that posttransplant *enterococcal* enrichment was accompanied by the depletion of *clostridia*, with a significant reduction in fecal butyrate in both pre-clinical models and patients with GVHD. This result was consistent with a recent prospective single-center study that included 201 patients undergoing allo-HSCT and 28 healthy donors (75), indicating that butyrate-producing *Clostridiales* diminished early in the course of allo-HSCT, which was involved in the increased acute GVHD severity and transplantation associated mortality. In addition, it has also been illustrated that patients suffering chronic GVHD exhibited lower circulating concentrations of the butyrate and propionate in day 100 plasma samples (76). Most recently, Hino and colleagues (77) analyzed the gut microbial signatures of 59 long-term survivors (1-21.7 years; median, 6.4 years) after allo-HSCT, and found that intestinal dysbiosis with decreased abundance of the butyrate-producing bacteria was present over a 10-year lifetime after discharge following allo-HSCT. Of them, only limited chronic GVHD patients displayed no depletion of butyrate-producing *Faecalibacterium*. Similarly, a study including 541 patients admitted for allo-HSCT conducted by Peled et al. (78) indicated that patients with the dominance of another butyrate-producing *Eubacterium limosum* also displayed a close association with the reduced risk of relapse or progression of disease (hazard ratio [HR], 0.82 per 10-fold increase in abundance; 95% confidence interval (CI), 0.71 to 0.95; *P* = .009). Of note, as a major energy source for intestinal epithelial cells (IECs), evidence indicated that butyrate could improve IECs junctional integrity, decrease apoptosis and mitigate GVHD in mice, and the loss of butyrate led to reduced degree of histone acetylation in IECs (79).

Apart from the associations with GVHD, gut microbiota also has potential implications for a variety of other toxic effects, including *γ-proteobacteria* domination predicting pulmonary complications after engraftment (80), and gram-negative intestinal domination predicting subsequent bloodstream infection (81), while a more stable gut microbial configuration protecting febrile neutropenia (82), and three distinct bacterial taxa (*Bacteroidetes*, *Lachnospiraceae*, and *Ruminococcaceae*) protecting post-engraftment *Clostridium difficile* infection (83). In addition, it has to be noted the critical roles of intestinal microorganisms beyond bacteria played in allo-HSCT. Of them, Legoff and colleagues (84), characterizing the dynamic evolution of gut virome in 44 recipients during allo-HSCT by metagenomics, found that the overall proportion of vertebrate viral sequences in the guts of all recipients increased progressively during the weeks following transplantation, and the RNA viral reads from picobirnaviruses were predictive of later occurrence of severe enteric GVHD of stage 2 or higher (HR = 2.66; 95% CI = 1.46-4.86; *P* = 0.001) through a time-dependent Cox proportional-hazards model. In addition, Rolling et al. (85) reported the fungal dysbiosis in a cohort of 156 patients during allo-HSCT by both longitudinal amplicon-based and culture-dependent analyses in 1279 fecal samples. Notably, *Candida parapsilosis* complex species, including *C. parapsilosis*, *C. orthopsilosis*, and *C. metapsilosis*, were the most common cultured fungi. Compared with those without pre-engraftment domination by *C. parapsilosis* complex species, patients with *C. parapsilosis* complex domination pre-engraftment exhibited a higher transplant-related mortality and worse OS.

### Effects of the gut microbial modulation on allo-HSCT recipients

On the basis of these findings, there has been tremendous interests in gut microbial modulation with the aim to improve the outcome of patients undergoing allo-HSCT (86–88), including antibiotics, diets, prebiotics, probiotics, and FMT.

#### Antibiotics

Recently, Severyn and colleagues (89) found that gut decontamination, by oral vancomycin-polymyxin B in patients undergoing allo-HSCT, might protect recipients against gut-derived bloodstream infection by reducing the prevalence of gut pathogens. Additionally, previous research has suggested that occurrence of GVHD after allo-HSCT in obesity mice could be mitigated by prophylactic antibiotic treatment (90). Despite this, great caution should be exercised when delineating the effects of antibiotics on allo-HSCT as increasing evidence has illustrated the detrimental roles of antibiotic administration in recipients. A retrospective research examined 857 allo-HSCT recipients from Shono and colleagues (91) reported that the use of antibiotics such as imipenem-cilastatin and piperacillin-tazobactam were linked with elevated GVHD-related mortality at 5 years. Through GVHD mice model, the authors further illustrated that imipenem-cilastatin treatment led to the loss of the protective mucus lining of the colon and intestinal barrier impairment, which might be explained by the enrichment of mucus-degrading *Akkermansia muciniphila*. Furthermore, an increased risk of patients occurring acute and intestinal GVHD by gut decontamination and prophylaxis has also been revealed in a meta-analysis of 18 references (92). Of note, the conflicting clinical results regarding the influence of antibiotics on the outcome of allo-HSCT might be explained by the different types of antibiotics and timing of treatment (87).

#### Diets, prebiotics, and probiotics

Dietary elements and nutritional strategies have been increasingly evaluated regarding the impact on allo-HSCT outcomes through modulating intestinal microorganisms (93). It has been revealed that mice with diet-induced obesity exhibited reduced survival associated with acute and severe gut GVHD, which was consistent with the inferior survival of allo-HSCT recipients with a high body mass index (BMI, >30) that presented decreased gut diversity and *Clostridiaceae* abundance (90). In addition, Li and colleagues (94) demonstrated the roles of tyrosine in acute GVHD murine models. Specifically, additional tyrosine supplementation could significantly prolong OS, alleviate symptoms at the early stage of acute GVHD by regulating the microbial composition and fecal metabolic phenotype. Likewise, prebiotic intake has also been observed to be an effective strategy for preventing acute GVHD in allo-HSCT in a prospective study (95). Namely, from pre-transplantation conditioning to day 28 after allo-HSCT, the combined administration of resistant starch and a commercially available prebiotics mixture (including glutamine, fiber, and oligosaccharide) decreased the incidence of all acute GVHD grades combined and of acute GVHD grades 2 to 4, and maintained the intestinal diversity and butyrate-producing bacterial population.

Compared with prebiotics, probiotics are viable microorganisms for healthy gut restoration. Importantly, the protective roles of the butyrate-producing *Clostridia* have been well demonstrated in preclinical allo-HSCT models (79, 96). Of them, Mathewson and colleagues (79) indicated that altering the indigenous microbiota, using the cocktail of 17 rationally selected *Clostridial* strains with the ability to produce high amounts of butyrate, could remarkably attenuate GVHD severity and improve survival. Furthermore, the safety and feasibility of another probiotic: *Lactobacillus plantarum* (LBP), have been also evaluated in children and adolescents undergoing allo-HSCT (97), and with no cases of LBP-bacteremia or LBP-associated severe adverse events recorded. Nevertheless, the safety and efficacy of probiotics in HSCT therapy remain elusive. For example, *Lactobacillus acidophilus* sepsis secondary to the excessive consumption of probiotic-enriched yogurt has been reported in a case with mantle cell lymphoma receiving HSCT (98).

#### Fecal microbiota transplantation

FMT refers to the transfer of fecal microbial content from a healthy donor to the intestine of a recipient (99), and represents a promising approach for the management of allo-HSCT patients (88), including alleviating infection of multidrug-resistant bacteria and GVHD, as well as promoting gut microbiota reconstitution. Bluestone and colleagues (100) reported that FMT displayed better safety and tolerance in three children developing recurrent *Clostridium difficile* infection after allo-HSCT, and one case did obtain successful clearance of *C. difficile* at follow-up 1 year 10 months after the FMT. Moreover, the safety and efficacy of FMT in the decolonization of multidrug-resistant bacteria, including vancomycin-resistant *enterococci* (n=2) or carbapenemase-producing bacteria (n=8), have also been presented in ten allo-HSCT patients with hematologic malignancies (101). Of them, seven of ten patients achieved decolonization and almost all patients without severe infectious events occurred during the first three months after FMT.

In a prospective, single-center, single-arm study enrolling 15 patients with steroid-refractory or steroid-dependent, acute or late-onset acute intestinal GVHD suffering allo-HSCT, van Lier et al. (102) found that ten of 15 subjects obtained a complete clinical response within 1 month after FMT, with a partial engraftment of donor microbial species, increased gut microbial α-diversity, and enrichment of butyrate-producing *Clostridiales* and *Blautia* species. As mentioned above, loss of intestinal diversity involves unfavorable allo-HSCT outcomes. Interestingly, FMT after allo-HSCT tends to be related to the improvement of recipients’ gut diversity that could be attributable to expansion of stool-donor taxa (103). In addition, it has been reported that autologous FMT (feces were provided by participants before the initiation of allo-HSCT), after microbiota-depleting antibiotic treatment, had the ability to boost microbial diversity and reestablished the commensal bacterial populations at the critical early immune reconstitution stage after allo-HSCT (104). Taken together, although FMT seemed safe and well-tolerated, further larger prospective studies are urgently required to deal with several safety concerns such as potential risks of infection upon FMT in these immunocompromised patients.

## Chimeric antigen receptor T cell therapy

CAR-T cell therapy stands at the novel forefront of current cancer therapy, which has demonstrated unprecedented responses in patients with high-risk hematologic malignancies, including lymphoma, leukemia, and multiple myeloma (105–108). CAR-T cell therapy involves genetically modified T cells that express specific CAR, followed by *in vitro* cell amplification and reinfusion back into the patient to eradicate tumors (109). Given the intimate interactions of gut microflora with human T cell function and anti-tumor immunity (110, 111), it is not unexpected that the interactions and potential mechanisms of gut microbiota with CAR-T cell therapy have begun to be investigated in recent years (112, 113). Of them, Uribe-Herranz et al. (114) illustrated that gut microflora could modulate the anti-tumor efficacy of adoptive T cell therapy, mediated by CD8α^+^ dendritic cells and IL-12, in the tumor-bearing mice model.

Although the success of CAR-T cell therapy, several obstacles, including CAR-mediated toxicities, CAR-T cell dysfunction, antigen loss, tumor heterogeneity, and disease relapse, have impeded the utility of CAR-T cell therapy. Therefore, biomarkers for the favorable prognostic identification of patients receiving CAR-T cells are urgently needed. Inspiringly, in a multi-center retrospective study including patients with B-cell lymphoma and leukemia, Smith et al. (115) found that exposure to antibiotics prior to CD19 CAR-T cell infusion was involved in significantly inferior survival and increased immune effector cell-associated neurotoxicity syndrome. Moreover, enrichment of certain members within the class *Clostridia*, including *Faecalibacterium*, *Ruminococcus*, and *Bacteroides*, were linked with day 100 complete response to CAR-T cell therapy. Similarly, Hu and colleagues (116) also revealed the significant differences in the abundance of *Bifidobacterium*, *Prevotella*, *Sutterella*, and *Collinsella* between multiple myeloma patients in complete remission and those in partial remission, and observed a higher abundance of *Bifidobacterium*, *Leuconostoc*, *Stenotrophomonas*, and *Staphylococcus* in patients with severe cytokine release syndrome. Altogether, despite the research on the role of intestinal microbiota in CAR-T cell therapy is still at the very earliest stages, these findings suggest the tremendous potential of gut microbiota as a non-invasive prognostic marker for CAR-T cell therapy, and provide a novel reference to alleviate CAR-T cell therapy-induced toxic effects and to improve therapeutic outcome by modulating the gut microbiota.

## Future perspectives and current challenges

Although major strides have been made toward the treatment of cancers, there remain many patients succumb to their disease (117). However, different from the stability of human genome, the modifiable nature of gut microbiota renders it a promising opportunity for cancer therapy (118). And meanwhile, there are emerging lines of evidence suggest the therapeutic potential of microbiotherapy by targeting the microbial flora. Among these, FMT appears central in the intervention options to restore microbial richness, as well as amend microbial dysbiosis and altered host-microbiota symbiosis related to cancer genesis and treatment (70). Moreover, utilizing bacteria strains or its proteins and peptides substances, including bacteriocins and toxins, as the anticancer agents on various cancers, termed bacteriotherapy, has also attracted salient attention, which can be employed alone or in conjunction with traditional therapies as an enhancer (119). Of interest, Montalban-Arques et al. (120) reported that oral supplementations of a mix of four *Clostridiales* species, namely *Roseburia intestinalis*, *Eubacterium hallii*, *Faecalibacterium prausnitzii*, and *Anaerostipes caccae*, outperformed anti-PD-1 therapy in mouse models of CRC and melanoma, which provided a strong preclinical foundation for exploring gut flora as novel stand-alone therapy against solid tumors. Additionally, despite the safety of probiotics in the management of cancer patients remains largely undefined, several gut next-generation probiotics such as *Faecalibacterium prausnitzii*, *Akkermansia muciniphila*, and *Bacteroides fragilis* exhibit their beneficial roles in supporting cancer therapy (121).

Most importantly, with mounting evidence of microorganisms colonizing tumors, synthetic biology approaches are being leveraged to improve the effectiveness of bacteriotherapy agents by repurposing bacteria. As an intelligent medicine, engineering bacteria are able to demonstrate autonomous control, sensing and responding to the internalization process, and subsequently releasing cargo (122). Furthermore, combinations of engineering bacteria with drug-loaded nanoparticles, monoclonal antibodies, oncolytic virus, and even CAR-T cells will also open charming options in oncology (123, 124).

Following the tremendous advances of cultivation-independent technologies and microbial analysis tools, the profiles of gut microbiota have been extensively revealed. While much attention has been given to gut bacteria, the contributions of other intestinal microorganisms such as viruses and fungi to cancer genesis and treatment also deserve further scrutiny. Furthermore, given the overlapped alterations of gut species across cancers (125, 126), future work is warranted to clarify the roles of gut microbiota-derived strategies, using machine learning algorithms, in precise risk stratification, prognostication, and therapeutic decision-making of cancer patients.

Although we believe that modulation of gut microflora will probably be the next vanguard in the management of cancer patients, however, several potential challenges should be mentioned. First, the exact mechanisms of action between gut dysbiosis and cancer genesis and therapy remain poorly characterized, and proofs of causation between them are still lacking. Therefore, continual efforts should be made to rationally select intestinal probiotics. On top of that, as a living body, the complexity of bacteria determines the hardships and risks such as biocontainment and safety concerns of transforming them into weapons to fight against cancers. Finally, considering the complex physiological conditions such as gastric acid and diverse enzymes that might digest or deactivate bacteriotherapy agents before they reach the action site, appropriate delivery route and dose of administration are also need to be investigated during clinical translations.

## Conclusion

In recent years, overwhelming pre-clinical and patient-oriented evidence supports a critical role of gut microbiota in cancer immunotherapies such as improving efficacy and mitigating toxicity, and the manipulation of gut microbiota confers a promising therapeutic strategy for the clinical management of malignancies as well. Currently, microbiotherapy for cancers is still in its infancy. With the formidable challenges notwithstanding, it deserves further mechanistic dissection by cellular and animal studies as well as validation with larger longitudinal clinical cohorts.

## Author contributions

MZ, ZL, and ZS conceived the study. ZS, HL, WS, and ZZ collected the related references and participated in the discussion. ZS and ZL drafted the manuscript and prepared the figures. MZ and ZS revised the manuscript. All authors contributed to the article and approved the submitted version.
